# The results of screw augmentation of acetabular cement spacers for the treatment of periprosthetic hip joint infection

**DOI:** 10.1186/s13018-020-01950-w

**Published:** 2020-09-29

**Authors:** Jing-Yao Jin, Taek-Rim Yoon, Kyung-Soon Park, Sheng-Yu Jin, Dong-Min Jung, Qing-Song Li

**Affiliations:** 1 Department of Orthopedic Surgery, Center for Joint Disease at Chonnam National University Hwasun Hospital, 322, Seo Yang-Ro, Hwasun-Eup, Hwasun-Gun, Jeonnam 519-809 Republic of Korea; 2 Department of Orthopedic Surgery, Center for Joint Disease, Affiliated Hospital of Yanbian University, Yanji, China

**Keywords:** Two stage, Infection, Dislodgement, Screw, Augmentation, Stability

## Abstract

**Introduction:**

Prosthesis of antibiotic-loaded acrylic cement (PROSTALAC) is widely used in two-stage revision arthroplasty in periprosthetic joint infection (PJI) after total hip arthroplasty (THA). In our institution, we encountered several cases of acetabular cement spacer dislodgement. The aim of this study was to compare the results of two-stage revision arthroplasties with antibiotic-loaded cement spacers with or without screws on the acetabulum for PJI.

**Patients and methods:**

This retrospective study included 44 patients who underwent a two-stage revision THA for PJI from June 2007 to May 2017. We divided the patients into two groups: group 1 consisted of 21 patients (21 hips) who underwent two-stage revision arthroplasty with screw augmentation, while group 2 consisted of 23 patients (23 hips) who underwent the same surgery without screw augmentation at the acetabular cement spacer. We compared the migration and dislodgement of the acetabular cement spacer between the two groups.

**Results:**

Before the second-stage surgery, there was less vertical migration of the cement spacer in group 1 compared to group 2 (1.2 mm vs 3.1 mm, *p* < 0.001). There was also less medial migration of the cement spacer in group 1 (0.6 mm vs 1.6 mm, *p* = 0.001). After the first stage, the mean Harris Hip score was significantly higher in group 1 than in group 2 (75 vs 65, *p* = 0.033). Cement spacer rotation or total movement out of the acetabular area occurred in six patients, all in group 2. After first stage reinfection occurred in two patients, one in each group.

**Conclusions:**

Screw augmentation to the acetabulum in the first-stage surgery provides better stability of acetabular antibiotic cement spacers without increasing reinfection rate.

## Introduction

Although its incidence rate is low, periprosthetic joint infection (PJI) is a serious and devastating complication of total hip arthroplasty (THA). It typically causes pain and disability, prolonging a patient’s hospital stay and increasing medical expenses [1]. There are numerous different clinical treatment options for PJI, such as resection arthroplasty (Girdle stone), single-stage revision, and two-stage revision [2–4]. Among these, two-stage revision offers a higher success rate and remains the gold standard. In two-stage revisions, antibiotic-loaded cement spacers can be used to deliver high-dose antibiotics via direct local delivery; they are also intended to provide stability, correct joint length, control pain, and mobilize the patient between stages to optimize function [5]. However, there are a number of complications associated with cement spacers, such as cement spacer dislocation, fracture, and reinfection [6–8].

Cement spacers can be broadly classified as being either articulating or non-articulating. An articulating antibiotic-loaded cement spacer allows early mobilization and efficient local antibiotic delivery and has therefore gained popularity. Numerous types of antibiotic-loaded cement spacers have been developed, some of which are currently commercially available. General spacer types include handmade +/− reinforcement rods, intra-operatively molded +/− reinforcement rods, prefabricated, and antibiotic-coated prosthesis (ACP) +/− polyethylene sockets. Special sub-types include mega-prosthesis for the setting of massive bone loss and partial-resections where a “hemi-cap” of antibiotic cement is placed onto the trunnion of a well-fixed femoral component [9]. In our institution, we have routinely used cement spacers for PJI patients since 2007. We encountered several cases of acetabular cement spacer dislodgement before we began using screws to augment cement stability to the acetabulum.

The purpose of this study was to compare the results of PJIs treated with two-stage revision hip arthroplasties with antibiotic-loaded cement spacers with and without screw augmentation to the acetabulum.

## Patients and methods

This retrospective study was performed on 44 PJI patients who underwent a two-stage revision arthroplasty with an antibiotic cement spacer from June 2007 to May 2017. All patients underwent THA prior to the first-stage surgery. A total of 21 patients (21 hips) who had an antibiotic-loaded cement spacer with one or two cancellous screws were defined as group 1 and the remaining 23 patients (23 hips), without screw augmentation, were defined as group 2. All patients were diagnosed with PJI by Parvizi et al. [10]. We compared the clinical and radiological results of the two groups. The study was approved by the Institutional Review Board of our hospital. Informed consent was obtained from each patient. The baseline data are detailed in Table 1. The bone defect of the acetabulum was evaluated by the Paprosky classification [11], which is detailed in Table 2. The exclusion criteria were patients with malignancy, metabolic bone disease, or previous osteomyelitis.

**Table 1:** Baseline data of with and without screw fixation

Variable	Group 1 (*n* = 21)	Group 2 (*n* = 23)	*p* value
	With screw	Without screw	
Gender (M/F)	16/5	14/9	0.276
Age	65.5 (range 32 to 78)	70.7 (range 53 to 86)	0.094
BMI	23.7 (range 19.1 to 34)	22.5 (range 17.8 to 27.8)	0.193
The duration from the primary operation to the first stage (month)	46.4 (range, 2-206)	69.4 (range, 2.6-209.9)	0.218
The duration from the first stage to the second stage (month)	4.5 (range, 1.7-14.6)	6.6 (range, 0.8-65)	0.463
First stage to the last follow-up (year)	5.9 (range 2.3 to 8.3)	5.5 (range 0.9 to 11)	0.535
Diagnosis before first-stage operation (all patients underwent THA)			
Femoral neck fracture	18	15	
Osteonecrosis of femoral head	3	8	

*M* male, *F* female, *BMI* body mass index, *THA* total hip arthroplasty

*Statically significant *p* value < 0.05

**Table 2:** The detail of bone defect evaluated by Paprosky classification

	Group 1 (*n* = 21)	Group 2 (*n* = 23)
I	10	10
IIa	4	5
IIb	2	2
IIc	1	1
IIIa	2	2
IIIb	2	3

**Fig. 1:**
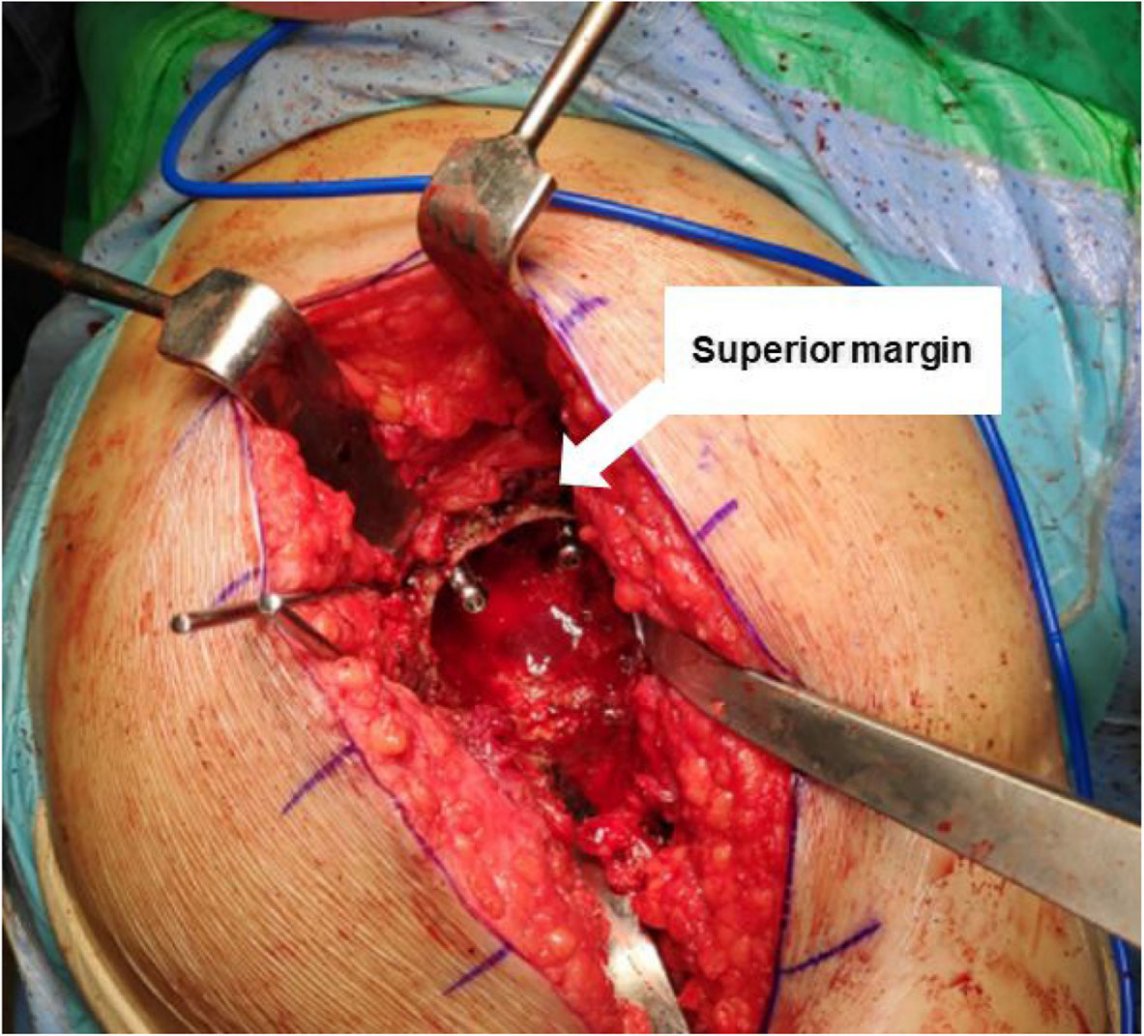
Intraoperative photography of insertion of 2 cancellous screws to the acetabulum

**Fig. 2:**
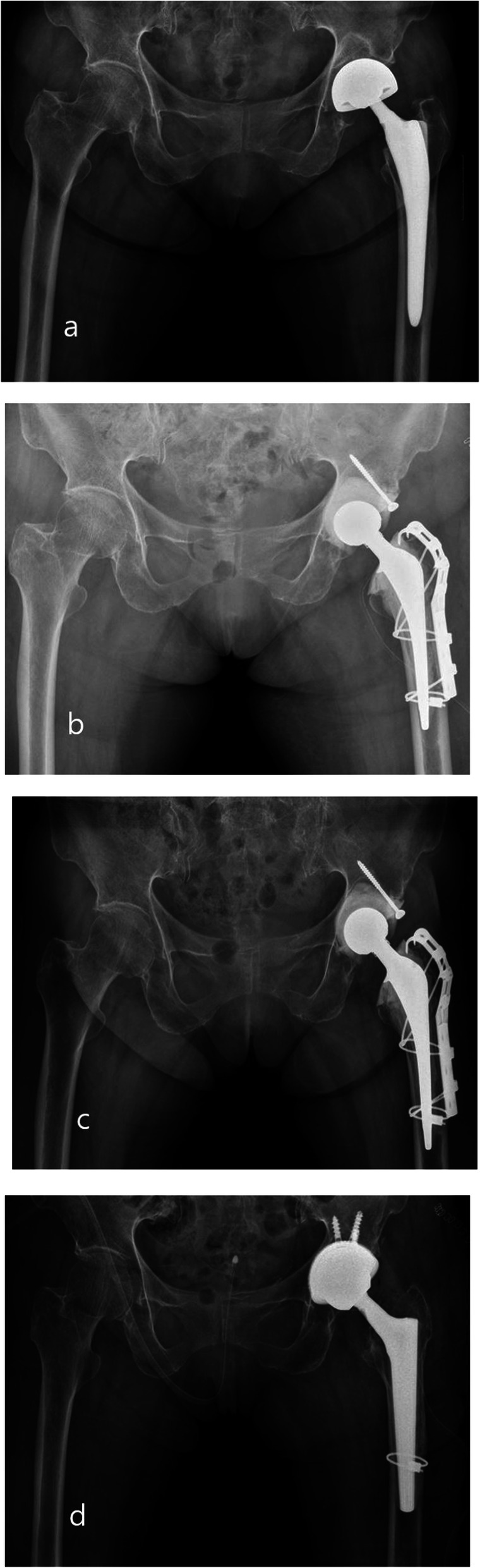
**a** A 66-year-old male patient did PROSTALAC combined with screw fixation due to PJI after THA. **b** Postoperative 1 week. **c** Postoperative 3 months, there was no change of vertical and horizontal distance after first stage immediately compared with the last follow-up. **d** When infection was free, the patient performed conversion THA

During all surgeries, antibiotic Simplex cement (Stryker, Allendale, NJ), impregnated with erythromycin, was used. For the acetabulum, one to two cancellous screws were fixed in combination with cement spacers at the posterosuperior area of the acetabulum (Fig. 1). The screw sizes ranged from 35 mm to 50 mm and the size used depended on the bone quality of the posterosuperior area of the acetabulum. A bolus of cement (1 pack, 41 g) was inserted into the acetabular cavity and molded into the shape of the cup. Screws were not used in group 2. For the femoral site, all patients had the original stem removed, and sterilized hospital-used stems (sterilized by ethylene oxide gas medical devices at 134 °C and stored in a sterile vacuum package) or CPT stems (Zimmer, Warsaw, UK) combined with a 28 mm or 32 mm metal head (Zimmer, Winterthur, Switzerland) were used with a collar of antibiotic-loaded cement (1 pack, 41 g) (Fig. 2). If the stem was not easy to remove, an extended osteotomy was added in the femur site and was cable fixed at the osteotomy site. The choice of antibiotic depended on the results of the preoperative bacterial culture, if this was positive. If the preoperative culture was negative, 2 g of vancomycin (Hanomycin®, Samjim pharmaceutical, Seoul, Korea) and 1 g of a piperacilin-tazobactam mixed antibiotic (Tazocin®, Wyeth Pharm, Seoul, Korea) were used on both the femoral components and acetabular cavities (Table 3). In our hospital, we have performed many revision procedures. The majority of the stems used in primary operation were collared femoral stems. The removed stems were stored for secondary use only for prosthesis of antibiotic-loaded acrylic cement (PROSTALAC) cases. Mainly, we use smaller stems so that there is adequate space for the antibiotic-loaded cement and to ensure the release of the antibiotics is effective.

**Table 3:** Isolated pathogens in the culture group

Isolated pathogen	Total number of cases	
	Group 1	Group 2
	(*n* = 21)	(*n* = 23)
MRSA	6	7
MRSE	4	7
*Bacillus* species		1
*Candida albicans*	4	2
*Escherichia coli*	3	2
*Kocuria rosea*	1	
*Pseudomonas aeruginosa*	1	1
*Klebsiella pneumonia*	1	1
No growth	1	2

A negative suction drain was used after wound closure and was retained until the drainage was clear and less than 20 ml per day. Intravenous antibiotics were given postoperatively for 4-6 weeks after the first-stage revision based on culture sensitivity in the culture-positive group. For the culture-negative group, postoperative antibiotics were selected in consultation with the Department of Infectious Disease at our institution. Broad spectrum cephalosporin antibiotics were used in one patient and vancomycin in two patients. Patients were allowed to move depending on their general condition postoperatively. Usually, after 2-3 days, the patients were allowed to move using a wheelchair or crutches. In some high-risk patients, mobilization was delayed until 4-5 days. The criteria for conversion to THA were (1) healing of the wound and sinus (if any), (2) return of conflict resolution policies (CRP) and equivalent series resistance (ESR) levels to normal, and/or (3) medical fitness for surgery [12].

In the second stage, a standard posterolateral approach was used, following the previous incision. During the second-stage surgery, our protocol was to use a cementless prosthesis on the femoral and acetabular components (Table 4). After the second stage, first-generation cephalosporin antibiotics were administered for 3–5 days postoperatively. Patients were followed at 1, 3, 6, and 12 months postoperatively and annually thereafter. Radiographs were obtained and measurements for total white blood cell (WBC), ESR, and CRP levels were repeated at each follow-up visit. It should be noted that all the cement spacers and screws were removed with ease. When removing the cement spacer, no bone ingrowth was detected at the surface of the cement spacer.

**Table 4:** Implants used in the first and second stage

	Group 1 (*n* = 21)	Group 2 (*n* = 23)
	With screw	Without screw
First stage		
Femur component		
Hospital sterilized used stem	19	20
CPT stem	2	3
Additional fixation		
Osteotomized of the femur (cables)	7	8
Prevention of the proximal femur site for poor bone quality (cables)	1	1
Grip plate ± femoral locking plate + cables (PF)	1	1
Second stage		
Acetabular component		
Delta PF cup with ceramic on ceramic	15	17
Ganz with metal on metal	6	6
Femur component		
Stem changed		
Wagner conical stem	10	10
Wagner conical long stem	11	8
Stem retained		
First stage revision stem (CPT stem)	0	5
Additional fixation		
Grip plate + cables (PF)	1	0

*PF* periprosthetic fracture

**Fig. 3:**
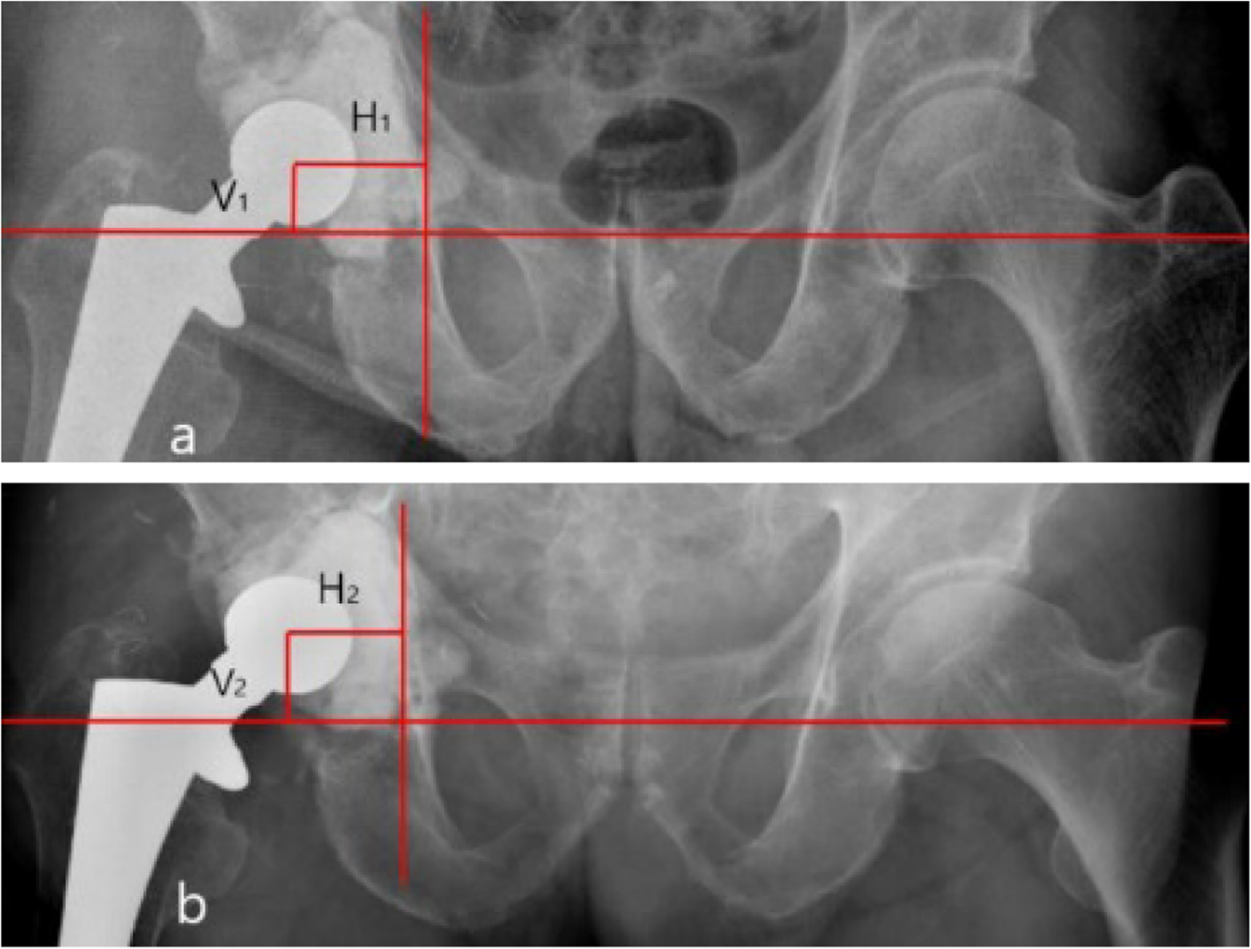
**a** Immediately postoperative X-ray of first stage. V is the vertical distance from femoral head center to the horizontal reference line. H is the horizontal distance from femoral head center to the vertical reference line. **b** Last follow up X-ray before second stage operation. Vertical migration is V2-V1 and horizontal migration is H1-H2

The primary outcome of this study was the grade of cement spacer migration. This was divided in two criteria; one is the distance that the femoral head center migrated in vertical or horizontal (medial) or both directions. Migration was measured by hip anteroposterior radiographic findings at final follow-up before the second-stage surgery and compared to the immediate radiograph. We defined the femoral head center as (C), a horizontal line connecting the inferior margin of tear drops was used as a reference line, and the distance from C to the horizontal line indicates vertical distance (V). Another vertical line, just medial of the tear drops, was used as a second reference line and the distance was measured from C to the vertical line as an indicator of horizontal distance (H) (Fig. 3). We calculated vertical migration of the cement spacer as V1-V2 and horizontal migration as H1-H2. The other one is the patients which cement spacer rotation or total movement out of the acetabular area. The number of patients who had a recurrent infection before or after the second stage surgery was determined. The clinical results were evaluated by the Harris hip score (HHS) [13] preoperatively, before the second-stage operation, and at final follow-up.

All data were analyzed using SPSS version 20.0 (SPSS Inc., USA). Student’s t test was used to analyze the normal distributed numerical variables. Pearson chi-square test or Fisher exact test was used to analyze the qualitative variables; *p* value ≤ 0.05 was considered to be statistically significant.

## Results

In our study, the follow-up duration was defined as the time from first stage to the final follow-up. The average follow-up duration was 5.9 years (range, 2.3 to 8.3 years) and 5.5 years (range, 0.9 to 11 years) in groups 1 and 2, respectively. The time from first stage to second stage was 4.5 months (range, 1.7 to 14.6 months) for group 1 and 6.6 months (range, 0.8 to 65 months) for group 2. The time from primary operation to the first stage was 46.4 months (range, 2 to 206 months) in group 1 and 69.4 months (range, 2.6 to 209.9 months) in group 2.

Surgical duration was calculated from incision to complete wound closure. There were no significant differences between surgical duration, intraoperative blood loss, or the amount of blood transfused in the first stage (Table 5). The HHS significantly increased between stages; however, between preoperative and final follow-up, there was no difference between the two groups. Prior to the second-stage surgery, the HHS was significantly different between the two groups (*p* = 0.033) (Table 6).

**Table 5:** Details of intraoperative parameters

	Group 1 (*n* = 21)	Group 2 (*n* = 23)	*p* value
	With screw	Without screw	
Operation time (min)	176 (range 110 to 255)	180.4(range 120 to 270)	0.757
Intraoperative blood loss (ml)	1094 (range 800 to 1500)	1147 (range 800 to 2000)	0.571
Transfusion (no. of units)	3.6 (range 2 to 5)	3.5 (range 2 to 6)	0.730

*Statically significant *p* value < 0.05

**Table 6:** The HHS in two groups

	Group 1 (*n* = 21)	Group 2 (*n* = 23)	*p* value
	With screw	Without screw	
Preoperative	39 (range 23 to 54)	43 (range 12 to 57)	0.236
Before second stage	75 (range 63 to 89)	65 (range 12 to 80)	0.033
Last follow-up	90 (range 78 to 95)	89 (range 83 to 93)	0.197

*HHS* Harris Hip score

*Statically significant *p* value < 0.05

Before the second-stage operation, there was less vertical migration of the cement spacer with screws compared to that without screws (1.2 mm vs 3.1 mm, *p* < 0.001). There was also reduced medial migration of the cement spacer in the group with screws compared to that in the group without screws (0.6 mm vs 1.6 mm, *p* = 0.001) (Table 7).

**Table 7:** Vertical and horizontal migration of cement spacer

	Group 1 (*n* = 21)	Group 2 (*n* = 17)	*p* value
Vertical migration (mm)	1.2 (range 0 to 2.6)	3.1 (range 0 to 7.3)	< 0.001
Horizontal migration (medial) (mm)	0.6 (range 0 to 2)	1.6 (range 0 to 3.4)	0.001

*Statically significant *p* value < 0.05

**Fig. 4:**
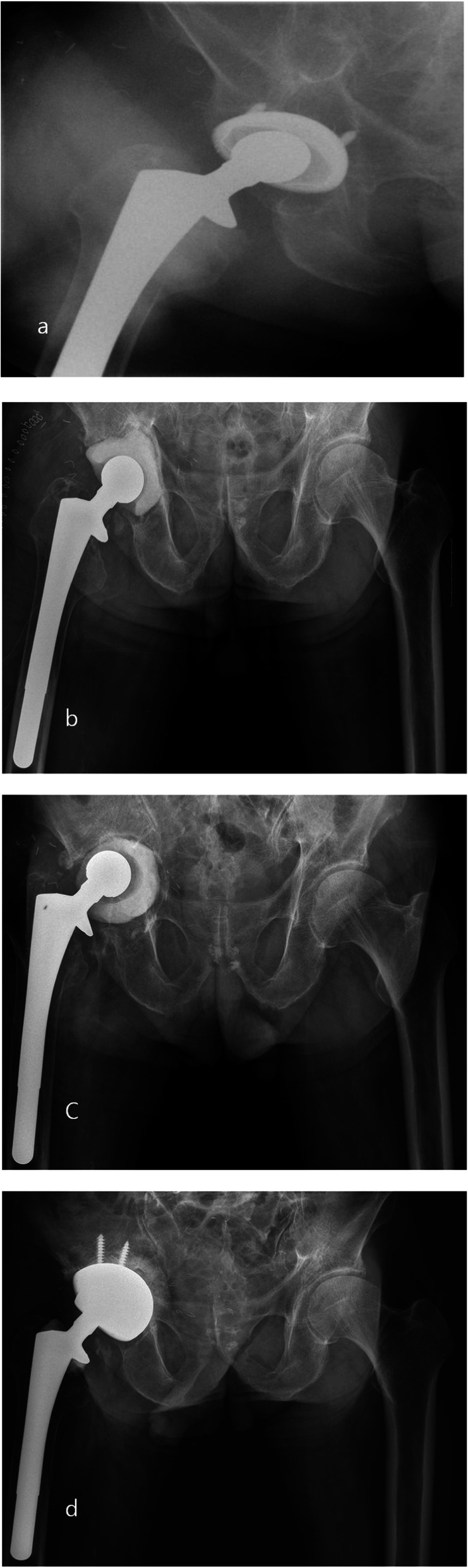
**a** A 62-year-old male patient did PROSTALAC with no screw fixation due to PJI after THA in case of an acetabular defect type PAPROSKY IIB. **b** Postoperative 1 week. **c** At 3 months follow-up, the cement spacer was rotated in the acetabular. **d** Finally, the patient performed revision THA

**Fig. 5:**
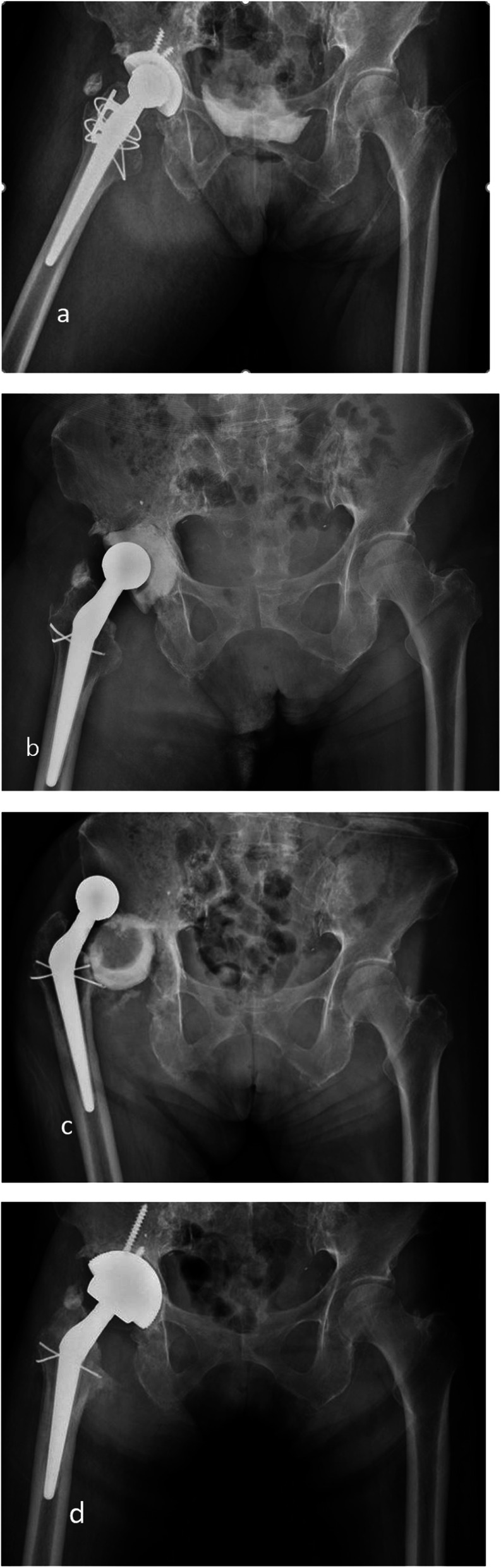
**a** A 68-year-old male patient did PROSTALAC with no screw fixation due to PJI after THA in case of an acetabular defect type PAPROSKY IIIA. **b** Postoperative 1 week. **c** At 3 months follow-up, the cement spacer was totally movement out of acetabular. **d** Finally, the patient performed revision THA

Only two patients, one in each group, underwent repeated radical debridement before re-implantation due to uncontrolled infections. Two patients had recurrent infection after the second stage, one patient from each group (*p* = 0.949). In the patient from group 1, infection occurred 2 years after the second stage; for the patient from group 2, infection occurred 1 year after the second stage. Both patients were infected by different bacteria compared to those involved in the first-stage infection. After the first stage, two patients had periprosthetic fractures at the femur site, one in each group. Femoral metal head dislocation occurred in one patient from group 1 and two patients from group 2 after the first-stage surgery. Cement spacer rotation (two hips) (Fig. 4) or movement out of the acetabular area (four hips) (Fig. 5) occurred in six patients, all from group 2 (Table 8).

**Table 8:** The complications after first stage

	Group 1 (*n* = 21)	Group 2 (*n* = 23)	*p* value
	With screw	Without screw	
Recurrence of infection	1	1	0.949
Periprosthetic fracture	1	1	0.949
Dislocation (metal head dislocation only)			
First stage	1	2	0.615
Cement spacer rotation or moved out from acetabular	0	6	0.022
Rotation	0	2	
Movement out	0	4	

*Statically significant *p* value < 0.05

## Discussion

Recurrence and repetitive infections may cause troublesome complications such as bone loss, poor soft tissue integrity, prolonged and complex operations, and physical and psychological disabilities. Because of the change in character of the remaining bone, the shear strength at the bone-cement interface is reduced and the cement-microinterlock necessary for long-term stability is lost [14, 15]. In particular, in the setting of infection, when acetabular bone loss is severe, the challenge for surgeons can be even greater due to the difficulty in implanting a stable cement spacer while keeping in mind the need for future surgery [16, 17].

Several techniques on how to manage bone loss and increase the stability of cement spacers in the setting of PJI have been described previously. Weiss et al. [17] treated PJI patients with serious medial acetabular wall deficiency with an antibiotic cement spacer combined with two acetabular screws. Although this was a case report, they noted that the acetabular screws played an important role in the stability of the cement spacer and this method could recreate a medial wall to prevent protrusion. They also noted that it is easy to construct during the first stage and easy to remove during the second stage. Rogers et al. [18] used cancellous screws and cement to treat 15 PJI patients, although these patients had supra-acetabular bone loss, they found utilizing screws and cement could improve acetabular coverage and reduce the risk of mechanical failure. Baker et al. [16] presented a technique with cancellous screws fixed in the supra-acetabular bone combined with cement and a polyethylene liner, creating a cement spacer in the setting of supra-acetabular osteolysis. Flahiff et al. [19] conducted an experimental study, which revealed the stability of a cement screw construct compared to an uncemented one. They reported that all cement screw constructs were significantly stronger than the uncemented controls with respect to the pull-out strength of the screw.

In our study, six patients experienced cement spacer rotation or total movement from the acetabulum and all of these patients were in group 2. All of these patients showed serious bone loss: two, Paprosky type IIIA; three, Paprosky type IIIB; and one, Paprosky type IIB. However, in group 1, there were no cases of cement spacer rotation or movement from the acetabulum, even though there were four cases of serious bone defects (two, Paprosky type IIIA; two, Paprosky type IIIB). Not all cases of cement spacer migration were in a vertical and horizontal direction in either of the two groups. In some patients, the degree of migration was less than 1 mm, although this could be due to a lack of radiographic standardization or varying exposure and rotation of the hip or because an unavoidable a source of error was introduced into our analysis. However, in group 1, the cement spacer was significantly less migrated in both the vertical (*p* = 0.003) and horizontal directions (*p* = 0.006). Moreover, all screws were removed smoothly with the cement spacer and there were no cases of screw fracture during the procedures.

Patient comfort was improved between stages by using screws, as reflected by HHS. We found no significant difference between preoperative and final follow-up HHS. Before the second stage, the HHS in group 1 was significantly higher than that in group 2. The mean HHS was 73 (range, 63 to 89) and 65 (range, 12 to 80) (*p* = 0.033) in groups 1 and 2, respectively. The majority of the difference seen in this study was in the improvement of pain, rather than in function. We believed screws could support the cement construction, improve the comfort of patients, and prevent cement migration by creating a stable, articulating antibiotic cement spacer.

Persistent infection or reinfection is a major complication of THA. The rate of reinfection is small, but it can cause financial and physical problems to patients. Degen et al. [20] considered that the control of infection may relate to the design of the spacer. Numerous types of antibiotic-loaded cement spacers have been developed. Although the results were satisfactory, a 6-11% reinfection rate has been reported [5, 8, 21]. Reinfection can potentially be because the causative organism was not eradicated completely, the re-infected organisms may have been present from the beginning but not initially isolated, or after the first stage, the patient was infected by different organisms. The authors considered that the PROSTALAC system, a metal-on-polyethylene combined with cement, could add another surface for bacterial adherence in the setting of infection. This may pose a theoretical additional risk of infection [4, 5, 8]. In this study, we excluded this potential risk factor. During the first stage, we did not use a metal-on-polyethylene liner at the acetabular side; nearly the entire spacer was coated with antibiotic-loaded cement, providing a better surface area for antibiotic delivery. After the first-stage operation, infections recurred in two cases, one in each group (*p* = 0.949), resulting in a reinfection rate of 5%, both with the same organisms. However, we could not determine the concentration of antibiotics on the acetabular and femur sides or if the reinfection was related to the screw augmentation; these aspects may require further research. Nonetheless, our results demonstrate that screw augmentation with acetabular cement spacer is safe and efficient.

## Conclusions

Screw augmentation in association with the use of antibiotic-loaded cement spacers in the first stage of a two-stage revision THA provides better stability of the cement spacer without increasing the reinfection rate.

## Data Availability

The datasets used and/or analyzed in the current study are available from the corresponding author on reasonable request.

## Abbreviations

PROSTALAC: Prosthesis of antibiotic-loaded acrylic cement
PJI: Periprosthetic joint infection
THA: Total hip arthroplasty
ACP: Antibiotic-coated prosthesis
CRP: Conflict resolution policies
ESR: Equivalent series resistance
WBC: White blood cell
C: Femoral head center
V: Vertical distance
H: Horizontal distance
HHS: Harris hip score
